# A comparative study of three-dimensional cone-beam CT sialography and MR sialography for the detection of non-tumorous salivary pathologies

**DOI:** 10.1186/s12903-023-03159-9

**Published:** 2023-07-08

**Authors:** Hélios Bertin, Raphael Bonnet, Aurélie Le Thuaut, Jean-François Huon, Pierre Corre, Eric Frampas, Emmanuelle Mourrain Langlois, Anne-Sophie Delemazure Chesneau

**Affiliations:** 1 Service de chirurgie maxillo-faciale et stomatologie, Nantes Université, CHU Nantes, Nantes, F-44000 France; 2 Nantes Université, UnivAngers, CHU Nantes, INSERM, CNRS, Nantes, CRCI2NA, F-44000 France; 3 Chirurgie maxillo-faciale et stomatologie, private practitioner, Clinique Brétéché, 3 rue de la Béraudière, Nantes, 44046 France; 4 Plateforme de méthodologie et biostatistique, direction de la recherche et de l’innovation, Nantes Université, CHU Nantes, Nantes, F-44000 France; 5 Nantes Université, CHU Nantes, Pharmacie, F-44000 France; 6 Nantes Université, Univ Tours, CHU Nantes, CHU Tours, INSERM, MethodS in Patients- centered outcomes and HEalth Research, SPHERE, Nantes, F-44000 France; 7 Nantes Université, UnivAngers, CHU Nantes, INSERM, Regenerative Medicine and Skeleton, RMeS, UMR 1229, Oniris, Nantes, F-44000 France; 8 Service d’imagerie médicale, Nantes Université, CHU Nantes, Nantes, F-44000 France

**Keywords:** Cone-Beam CT, Sialography, MR sialography, Salivary Duct pathology

## Abstract

**Background:**

Imaging of the salivary ductal system is relevant prior to an endoscopic or a surgical procedure. Various imaging modalities can be used for this purpose. The aim of this study was to compare the diagnostic capability of three-dimensional (3D)-cone-beam computed tomography (CBCT) sialography versus magnetic resonance (MR) sialography in non-tumorous salivary pathologies.

**Methods:**

This prospective, monocenter, pilot study compared both imaging modalities in 46 patients (mean age 50.1 ± 14.9 years) referred for salivary symptoms. The analyses were performed by two independent radiologists and referred to identification of a salivary disease including sialolithiasis, stenosis, or dilatation (primary endpoint). The location and size of an abnormality, the last branch of division of the salivary duct that can be visualized, potential complications, and exposure parameters were also collected (secondary endpoints).

**Results:**

Salivary symptoms involved both the submandibular (60.9%) and parotid (39.1%) glands. Sialolithiasis, dilatations, and stenosis were observed in 24, 25, and 9 patients, respectively, with no statistical differences observed between the two imaging modalities in terms of lesion identification (p_1_ = 0.66, p_2_ = 0.63, and p_3_ = 0.24, respectively). The inter-observer agreement was perfect (> 0.90) for lesion identification. MR sialography outperformed 3D-CBCT sialography for visualization of salivary stones and dilatations, as evidenced by higher positive percent agreement (sensitivity) of 0.90 [95% CI 0.70–0.98] vs. 0.82 [95% CI 0.61–0.93], and 0.84 [95% CI 0.62–0.94] vs. 0.70 [95% CI 0.49–0.84], respectively. For the identification of stenosis, the same low positive percent agreement was obtained with both procedures (0.20 [95% CI 0.01–0.62]). There was a good concordance for the location of a stone (Kappa coefficient of 0.62). Catheterization failure was observed in two patients by 3D-CBCT sialography.

**Conclusions:**

Both imaging procedures warrant being part of the diagnostic arsenal of non-tumorous salivary pathologies. However, MR sialography may be more effective than 3D-CBCT sialography for the identification of sialolithiasis and ductal dilatations.

**Trial registration:**

NCT02883140.

**Supplementary Information:**

The online version contains supplementary material available at 10.1186/s12903-023-03159-9.

## Background

Non-tumorous salivary gland diseases are common in adults and include salivary stones, ductal strictures, anatomical abnormalities, and chronic inflammation. Sialolithiasis is the main cause, representing 60–70% of all cases of obstructive disease, followed by stenosis in 15–25% [1–3]. Various imaging tools have been developed for the diagnosis of such diseases and to generate an accurate map of the salivary ducts [4, 5]. Regarding sialolithiasis, the size, location, mobility, and shape of the stone are considered prognostic factors that influence the treatment outcomes [6]. These criteria are part of the treatment algorithms for salivary calculi, and they determine the type of removal technique to be used [7, 8]. The same approach based on location, type, and length is used for the diagnosis and treatment of salivary stenosis [9].

Thanks to a direct opacification of the salivary ducts, conventional sialography has long been the gold standard for diagnosing non-tumorous salivary diseases [4, 5, 10]. By allowing detailed exploration of the ductal tree and late evacuation imaging, it provides assessment of salivary gland function and obstructive conditions [11, 12]. Three-dimensional (3D)-cone-beam computed tomography (CBCT) sialography is becoming increasingly common as it provides fast high spatial resolution 3D images of the ductal system [13, 14]. Several studies have shown the superiority of 3D-CBCT sialography compared to conventional sialography for visualization of ducts up to the 6th branch of division and for detection of stenosis and sialolithiasis [15–18]. It remains an irradiating and invasive procedure that cannot be used as a first-line examination, and it should be limited to cases amenable to routine ultrasonography and/or CT scan with obvious salivary symptoms, or prior to a sialendoscopic procedure [18]. MR-sialography represents an alternative for exploration of salivary ductal diseases [11, 19–22]. Its advantages stem from the non-ionizing nature of the procedure and the absence of catheterization thanks to the analysis of spontaneous contrast of saliva. However, it suffers from poor accessibility, high cost, long acquisition times, and lower sensitivity for exploration of the proximal salivary branches (i.e., intraglandular) [23, 24]. By exploring the inflammatory signal of the salivary glands, MR sialography is also a valuable imaging tool for evaluation of the severity of lesions in chronic inflammatory diseases including primary Sjögren’s syndrome (pSS) [25]. Comparative studies of MR sialography versus conventional 2D sialography have highlighted the ability of this technique to diagnose most salivary gland diseases, including sialolithiasis and stenosis, but also sialectasis and destruction of the glandular parenchyma associated with chronic diseases [11, 19]. High-resolution T2-weighted 3D gradient-echo and ultrafast T2-weighted spin-echo (SE) sequences appear to be more suitable for salivary exploration. However, conventional sialography provides better resolution and remains superior to MR sialography for documentation of third-order branches, proximal ducts, concretions, and changes in sialodochitis [19]. To our knowledge, there have been no objective comparative studies to date of MR sialography and 3D-CBCT sialography.

The purpose of this study was to compare the overall diagnostic outcomes of 3D-CBCT sialography versus MR sialography for the detection of sialolithiasis, ductal dilatation, and ductal stenosis, and the outcome measures for each of these three diseases. The null hypothesis predicts that there are no statistically significant differences between the two techniques in overall or disease-specific outcomes.

## Methods

This prospective, monocenter, pilot study included patients over 18 years of age (no upper age restriction) with suspicion of obstructive or inflammatory disease of a parotid or a submandibular gland based on the medical history and clinical examination. The exclusion criteria comprised having a known allergy to iodinated contrast agent, known or suspected pregnancy, lesion of the oral mucosa preventing catheterization, relative contraindication to MRI (prosthesis or metallic foreign body and/or claustrophobia), and signs of a salivary tumor (salivary mass, adenopathy, facial paralysis). Patients with a head and neck scan in the previous 6 months, either by conventional or three-dimensional sialography, were also excluded from this study. For each patient, the following clinical data were collected prior to the imaging procedures: age, gender, medical history, affected gland, side, complaints (swelling, salivary colic, or infection), and clinical parameters (palpable stone, increase in glandular volume, and the presence or absence of saliva at the ostium). After being provided information regarding the study and obtaining informed written consent, the included patients underwent MR sialography followed by 3D-CBCT sialography on the same day. The study was approved, and ethical approval was obtained from our institutional review board CPP Ouest IV-Nantes (reference 17/16, first registration on 18/03/2016, acceptance on 11/05/2016). This study was registered on ClinicalTrials.gov (number NCT02883140) on 30/08/2016. The STARD checklist for diagnostic studies [26] and the STROB items for observational studies [27] are provided in the Supplementary Materials.

### MR sialography

MR imaging was performed on a 3T scanner (INGENIA 3T, Philips Medical Systems, Amsterdam, Netherlands) using a head and neck coil. The patients were told not to eat or drink in the hour preceding the scan. The patients were positioned in dorsal decubitus and instructed not to swallow saliva during the acquisition times.

For sialo-MR sequence acquisitions, three-dimensional (3D) T2 DRIVE (DRIVen Equilibrium) with sagittal fat-suppressed turbo spin-echo (TSE) sequences were obtained with the following parameters: repetition/echo time (TR/TE) 2000/237 ms; matrix 376 × 267; field of view (FOV) 150 × 150; slice thickness 2 mm; interpolation 0.4; voxel 0.4 × 0.5 × 0.4, number of slices 94; spectral presaturation inversion recovery (SPIR); and scan time 4.34 min.

Conventional MR sequences were performed for diagnostic purposes in patients as part of routine care, including axial T2 TSE: TR/TE 2336/80 ms; TSE factor 14; NSA 1.5; matrix 416 × 320; FOV 180 × 180; slice thickness 2 mm; space 0.3; voxel 0.3 × 0.56 × 3 mm; number of slices 24; and acquisition time 2.48 min. Coronal T2 TSE: TR/TE 2726/80 ms; TSE factor 14; NSA 2; matrix 360 × 273; FOV 180 × 180; slice thickness 2 mm; space 0.3; voxel 0.5 × 0.66 × 3 mm; SENSE factor 1.4; number of slices 27; and acquisition time 2.38 min. Axial T1 TSE sequences: TR/TE 662/10 ms; factor TSE 5; NSA 1; matrix 364 × 300; FOV 200 × 180; slice thickness 3 mm; voxel 0.55 × 0.66 × 3 mm; number of slices 23; and acquisition time 2.47 min.

### 3D-CBCT sialography

The 3D-CBCT sialography procedure was performed by two senior maxillofacial surgeons (H.B. and R.B.), as previously described [28]. Briefly, after locating the salivary duct ostium, 0.5 mL of high-concentration, water-soluble, iodinated contrast product (HEXABRIX 320®, 320 g/L; Guerbet, France) was injected under standard aseptic conditions using a lachrymal cannula (25G Moria® L12 mm; MORIA Inc., Doylestown, PA, USA) until the patient felt fullness of the gland. The contrast medium was immediately maintained in the gland by placing a microsurgical clamp (Biover® TKM2, Hergiswil, Switzerland) on the ostium of the Wharton’s duct or with a plastic straight Halstead clamp placed on the ostium of the Stensen’s duct. Local anesthetic was administered systematically near the ostium. Image acquisition was performed immediately after injection, with the patient in a seated position, using a wide-field CBCT device (NewTom VGi, QR, Verona, Italy). Front and profile scout views were obtained first. Specific exposure parameters were used to limit the amount of irradiation: a reduced field of view of 75 × 120 mm focused on the symptomatic gland and limited to 110 kV. The tube current was adjusted automatically by the machine, while the exposure time was selected by the user (regular zoom mode). For each patient, we collected the dose-area product (DAP) provided by the CBCT device, as well as early adverse effects of cannulation.

### Image analysis

The imaging data were anonymized and archived using imaging software (CARESTREAM View PACS v. 11.3; Carestream Health, Inc., Rochester, NY, USA). Analysis of 3D-CBCT sialography was performed using maximal intensity projection and multiplanar reconstruction (MPR) with 0.25-mm cuts, isotropic voxel size and image pixels 492 × 492. MRI scans were analyzed on a high-resolution visualization workstation with native 2D image analysis (viewer mode) for TSE/T1/T2 sequences, and MPR for the 3D T2 MR-sialography sequences. Images were analyzed twice by two radiologists experienced in head and neck radiology (A.S.D. and E.M.L.), in separate viewing sessions (wash-out period of at least one month) and following a standardized pathway. The readers were blinded to the clinical data. A reading standardization session was performed on ten cases before starting the analyses. The analysis was based on the ability of the examination to identify a salivary disease including sialolithiasis (L), stenosis (S), or ductal dilatation (D) (primary endpoint). On MR sialography, saliva manifested as a high signal intensity and the salivary glands appeared as a low signal intensity, while only the salivary canal was opacified by the contrast medium in 3D-CBCT sialography. Sialolithiasis appeared in MR sialography as round or oval hypointense structures surrounded by hyperintense saliva, while the presence of a dense calcified concretion could be seen on 3D-CBCT sialography. Ductal stenosis displayed a signal or opacification interruption within the salivary ducts, and dilatations appeared as sialectasis in the ductal salivary system. Salivary diseases were classified according to the Marchal et al. classification (Table 1) [29]. Regarding sialolithiasis, the original classification was modified to reflect the degree of ductal obstruction: a floating calculus was defined as a calculus with a diameter significantly smaller than the salivary duct, a calculus was embedded if it remained permeable to the passage of contrast medium on 3D-CBCT sialography or to saliva (hyperintense signal) on MR-sialography despite a large size. Finally, an obstructive calculus did not allow upstream opacification with 3D-CBCT sialography and/or was associated with the absence of downstream saliva signal with MR-sialography. The other measured parameters were the precise location of the lesion in the division branches, the size of the lesion, and the last division branch visualized. Because individual readings showed little divergence, the data regarding the identification of the salivary diseases, as well as the location, the number, and the size of the lesions were averaged for the two readers to compare the two imaging modalities.

**Table 1:** Classification of salivary diseases according to the modified Marchal classification

Classification of sialolithiasis	
**L**_**0**_	Absence of sialolithiasis
**L**_**1**_	Floating calculus
**L**_**2**_	Embedded calculus
**L**_**3**_	Obstructive calculus
Classification of stenosis	
**S**_**0**_	Absence of stenosis
**S**_**1**_	Diaphragm-like intracanal stenosis (single or multiple)
**S**_**2**_	Single duct stenosis
**S**_**3**_	Multiple duct stenosis
**S**_**4**_	Generalized stenosis (‘dead tree’ appearance)
Classification of dilatations	
**D**_**0**_	Absence of dilatation
**D**_**1**_	Single duct dilatation
**D**_**2**_	Multiple duct dilatations
**D**_**3**_	Generalized dilatations

### Statistical analysis

For a sialolithiasis detection rate of 100% by sialography [16] and 80% by MR sialography [24], at least 37 patients were needed for this study (power: 80%). The statistical analysis was performed using SAS software, version 9.4 (SAS Institute Inc.). The intra- and the inter-observer agreement (IOA) was tested using the weighted Kappa coefficient: An IOA ≥ 0.8 was considered to be perfect, IOA between 0.6 and 0.8 was strong, IOA between 0.4 and 0.6 was moderate, and an IOA < 0.4 was considered as poor. McNemars’s *χ*^2^ test was used to determine differences between the two imaging modalities with regard to the same outcomes. A Wilcoxon signed-rank test was used to compare the last branch of division that could be visualized, the number of sialolithiasis, and their location in the salivary tree. A p-value of less than 0.05 was taken to indicate statistical significance. In the absence of a gold standard procedure (i.e., third imaging examination, sialoendoscopy, or surgical procedure), each imaging procedure was compared to the other, assuming that a lesion detected by one modality confirmed the presence of the disease. As a result, unbiased estimates of “accuracy”, “sensitivity”, and “specificity” cannot be calculated and the terms should not be used [16]. Therefore, the diagnostic properties of each imaging modality were estimated as an agreement of one imaging compared to the other, defining the positive percent agreement corresponding to the ratio between the number of positive cases with a radiological exam and the total number of patients with a positive examination.

## Results

### Clinical data

**Fig. 1:**
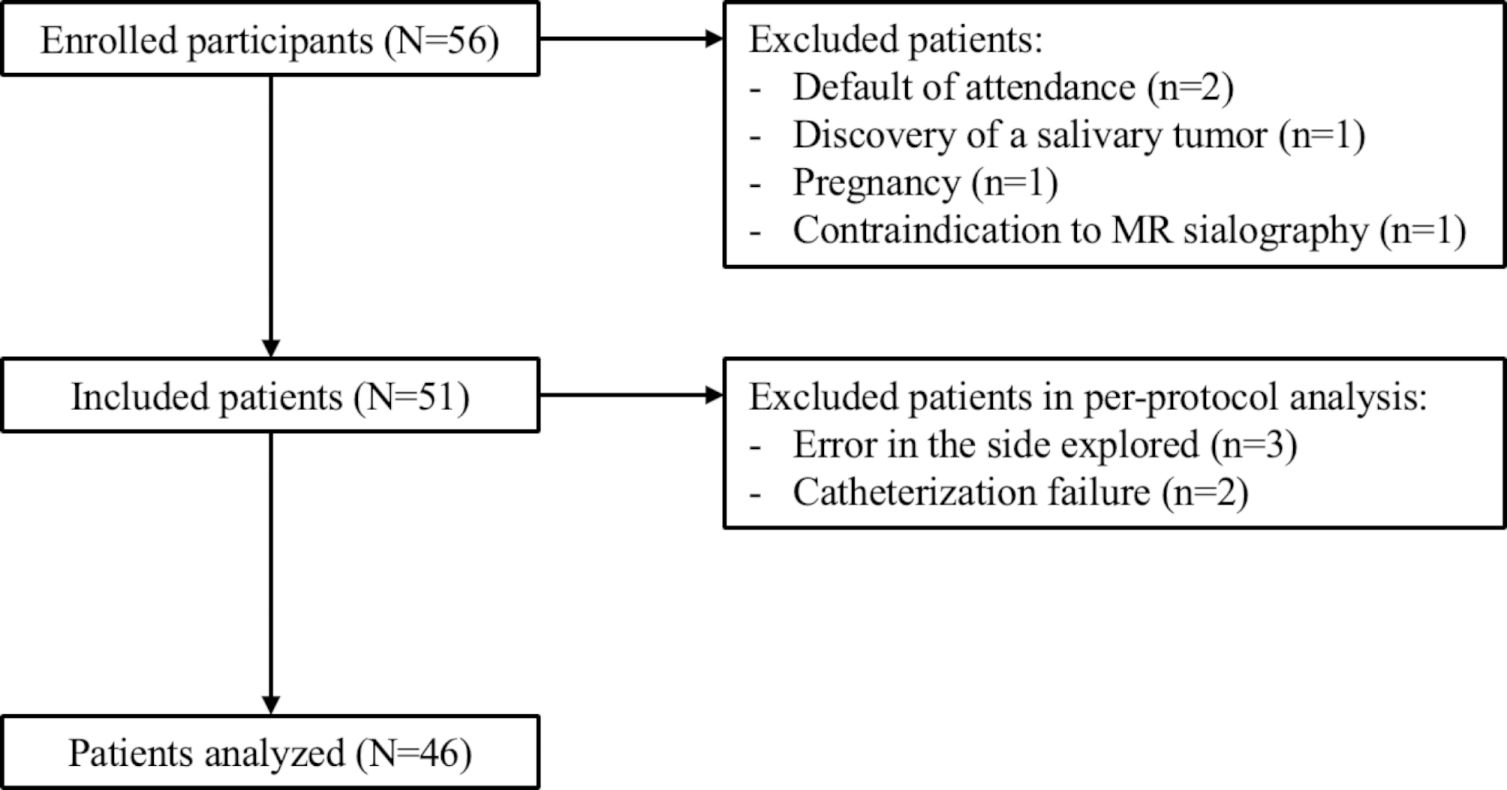
Flowchart of the selection of the participants

Between June 2016 and November 2017, 56 patients were enrolled in the study. Five patients were primarily excluded because of failing to present for one of the two procedures (n = 2), one patient was excluded due to an incidental finding of a tumor lesion by MRI, one patient for an ongoing pregnancy, and one for relative contraindication to MRI. Five patients were secondarily excluded in the per-protocol analysis due to a discrepancy in the side explored between the two examinations (n = 3), these patients exhibited an atypical symptomatology with multifocal salivary involvement, and due to catheterization failure for 3D-CBCT sialography (n = 2) (Fig. 1). The final analysis included 46 patients, with a mean age of 50.1 years. Six patients suffered from systemic disease, one had previously received radioactive iodine treatment, and one patient had already undergone a Wharton papillotomy. The symptomatology involved the submandibular gland in 28 patients and the parotid gland in 18 patients, with multifocal involvement in 6 cases. Most of the patients presented with obstructive symptoms consisting of salivary gland swelling in 36 cases, pain in 32 cases, and infection in 15 patients. One-fourth of the patients reported atypical symptoms including obstructive symptoms not related to meals, pain projected outside of the salivary area, or scratching occurring at mealtimes. The patient characteristics are presented in Table 2.

**Table 2:** Epidemiologic and clinical characteristics of the patients. n, number of patients; S.D., standard deviation

Patient characteristics	
Age (years), mean ± S.D. (range)	50.1 ± 14.9 (20.8–81.3)
Gender: Females/Males, n (%)	30 (65.2%)/16 (34.8%)
Affected gland:	
Submandibular gland, n (%) Parotid gland, n (%) Multifocal involvement, n (%)	28 (60.9%) 18 (39.1%) 6 (13.0%)
Symptoms:	
Swelling Pain Infection Atypical signs	36 (78.3%) 32 (69.6%) 15 (32.6%) 12 (26.1%)
Progression of symptoms:	
Less than 14 days 2–12 weeks More than 3 months	1 (2.2%) 3 (6.5%) 42 (91.3%)
Clinical examination:	
Palpable sialolithiasis Increased volume of the gland Saliva at ostium	14 (30.4%) 22 (47.8%) 40 (86.8%)

### Intra- and inter-observer agreement

The intra-observer agreement was perfect as estimated at 1.00 (1.00–1.00) for both radiologists, for both imaging procedures, irrespective of the identification of abnormalities or the last branch visualized. The IOA was perfect with both imaging modalities for the identification of sialolithiasis, stenosis, and dilatations (Table 3). A perfect agreement was observed between the two observers for visualization of the last branch of the canal division, except for the Wharton’s duct with MR sialography, for which a strong correlation was observed (Table 3).

**Table 3:** Inter-observer agreement for the two imaging modalities

Outcomes	Interobserver agreement	
	*3D-CBCT sialography*	*MR sialography*
Primary outcome: identification of diseases		
Sialolithiasis Stenosis Dilatation	0.96 (0.87–1.00) 0.90 (0.70–1.00) 0.95 (0.86–1.00)	1.00 (1.00–1.00) 0.90 (0.70–1.00) 0.96 (0.88–1.00)
Secondary outcome: last salivary branch visualized		
Stensen’s duct Wharton’s duct	0.80 (0.61–0.98) 0.87 (0.76–0.98)	0.78 (0.55–1.00) 0.61 (0.40–0.82)

### Identification of a salivary disease (primary outcome)

The two imaging modalities resulted in concordant interpretations for 38 of the 46 subjects. Of these, 22 were considered to be abnormal and 16 were interpreted as being normal. In two patients with abnormal examinations by 3D-CBCT sialography, the MR sialography failed to detect a ductal disease but produced an inflammatory signal and revealed intraglandular cysts related to pSS. The abnormal findings are summarized in Table 4.

**Table 4:** Comparison of the two imaging modalities regarding the primary and secondary outcomes. N, number of patients; n, number of lesions (in case of multiple salivary diseases in a given patient); IG, intraglandular

	*3D-CBCT sialography*	*MR sialography*	*Both modalities*	*p*
Identification of a salivary disease				
Normal examination, N (%) Abnormal examination, N (%)	20/46 (43.5) 26/46 (56.5)	20/46 (43.5) 26/46 (56.5)	16/46 (34.8) 22/46 (47.8)	0.83
Type of salivary disease				
Sialolithiasis, N (%) Stenosis, N (%) Dilatation, N (%)	20/24 (83.3) 5/9 (55.6) 19/25 (76.0)	22/24 (91.7) 5/9 (55.6) 23/25 (92.0)	18/24 (75.0) 1/9 (11.1) 16/25 (64.0)	0.66 0.63 0.24
Characteristics of sialolithiasis				
Classification, n				
L_1_ L_2_ L_3_	7/29 14/29 8/29	6/32 21/32 5/32	1/36 12/36 4/36	0.84 0.26 0.40
Sialolithiasis location, n				
Main canal Secondary branch (IG) Tertiary branch (IG)	19/29 9/29 1/29	25/32 5/32 2/32	16/36 3/36 1/36	0.41 0.26 0.93
Characteristics of stenosis				
Classification, n				
S_1_ S_2_ S_3_ S_4_	0/5 0/5 3/5 2/5	1/6 1/6 4/6 0	0/11 0/11 1/11 0/11	0.92 0.92 0.68 -
Location, n				
Main canal IG	4/5 1/5	4/6 2/6	0/11 0/11	0.85 0.85
Characteristics of dilatations				
Classification, n				
D_1_ D_2_ D_3_ D_4_	14/19 4/19 0/19 1/19	17/23 3/23 1/23 2/23	11/25 2/25 0/25 1/25	0.73 0.78 0.92 0.86
Location, n				
Main canal Secondary branch (IG) Tertiary branch (IG)	9/19 5/19 5/19	14/23 6/23 2/23	4/25 3/25 0/25	0.57 0.73 0.26

**Fig. 2:**
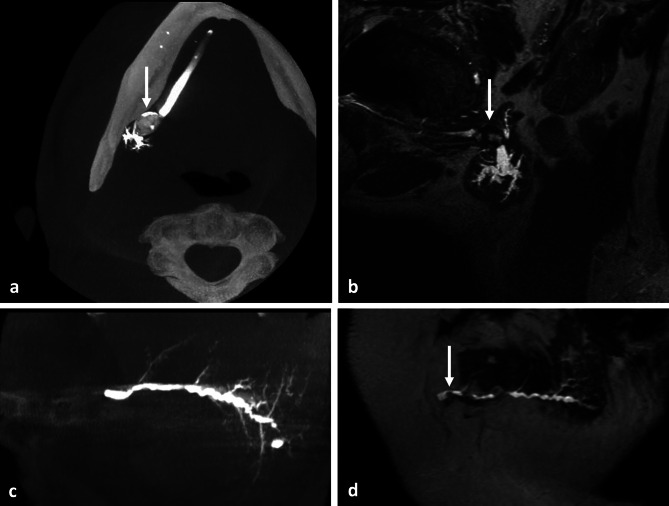
Identification of sialolithiasis. Large salivary stone (white arrow) located in the proximal third of the right Wharton’s duct, visible as calcified concretion within an opacified salivary duct with 3D-CBCT sialography in maximal intensity projection (MIP) axial view (**a**), and as a strong hyposignal with upstream hyperintense ductal dilatation in MR sialography in MIP sagittal oblique view (**b**). Case of discordance between the two imaging examinations with a distal sialolithiasis of the left Stensen’s duct undetected on the sagittal MIP view in 3D-CBCT sialography (**c**) and diagnosed on the sagittal MR sialogram (**d**)

3D-CBCT sialography and MR sialography allowed detection of 20 and 22 sialolithiasis, respectively (p = 0.66) (Fig. 2a and b). Six cases were discordant regarding the diagnosis of sialolithiasis: 4 stones located in the Stensen’s duct (mean size of 4.25 ± 2.01 mm) were only detected by MR sialography (Fig. 2c and d). Two sialolithiasis were diagnosed exclusively by 3D-CBCT sialography, with one located in a secondary branch of the parotid gland and the other in the Wharton’s duct. MR sialography outperformed 3D-CBCT sialography for visualization of sialolithiasis, as evidenced by a higher positive percent agreement: 0.90 [95% CI 0.70–0.98] vs. 0.82 [95% CI 0.61–0.93].

**Fig. 3:**
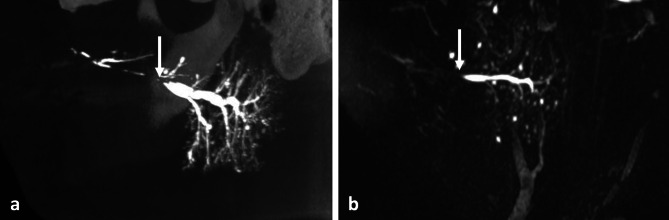
Imaging of the left parotid gland showing a stenosis (white arrow) of the pre-masseteric portion of the Stensen’s duct visible in MIP sagittal view with 3D-CBCT sialography (**a**) and MR sialography (**b**), upstream ductal dilatation is observed in sialography, as for intraglandular multiple cystic dilatations in both imaging modalities

**Fig. 4:**
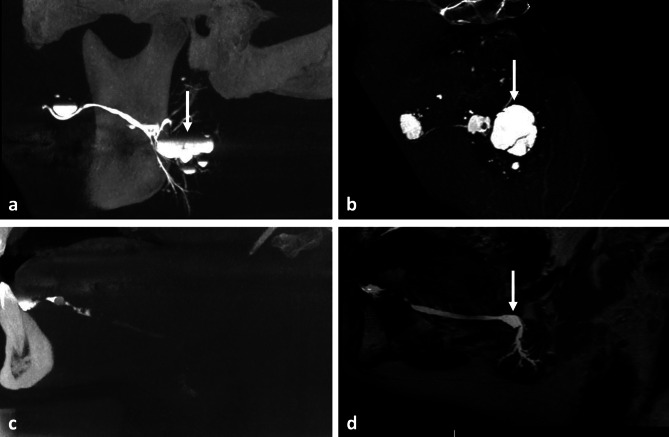
Exploration of salivary dilatations. Analysis of the left parotid gland revealing the presence of sialoceles of the anterior part of the main duct and in an intraglandular location (white arrow) with 3D-CBCT sialography in sagittal oblique MIP view (**a**) and MR sialography in native sagittal view (**b**). Case of a patient presenting with a distal obstructive sialolithiasis of the right wharton’s duct, with no upstream opacification in 3D-CBCT sialography in sagittal view (**c**), and with proximal dilatation of the salivary ducts visible on the MR sialography in MIP sagittal view (**d**)

Stenosis and ductal dilatation were observed in 9 and 25 cases, respectively, by one or the other radiological examination, with no statistical differences between the procedures for the identification of these salivary diseases (p_1_ = 0.63 and p_2_ = 0.24, respectively). The procedures were discordant regarding the diagnosis of stenosis, with only one case being diagnosed by both modalities (Fig. 3a and b). Regarding ductal dilatations, both modalities were able to detect such salivary abnormality in 16 patients (Fig. 4a and b), while 9 were discordant (6 were identified only by MR sialography and 3 only by 3D-CBCT sialography) (Fig. 4c and d). MR sialography and 3D-CBCT sialography displayed the same positive percent agreement regarding salivary stenosis: 0.20 [95% CI 0.01–0.62]. With regard to ductal dilatation, MR sialography outperformed 3D-CBCT sialography, with positive percent agreement of 0.84 [95% CI 0.62–0.94] vs. 0.70 [95% CI 0.49–0.84], respectively.

### Secondary outcomes

Multiple sialolithiasis were identified in 9 patients, amounting to 36 analyzable calculi (29 identified by 3D-CBCT sialography and 32 by MR sialography). Most of the stones were classified as being embedded in the salivary duct (L2). There was no difference between the two examinations for the classification of the calculi (Kappa coefficient: 0.76 [95% CI 0.65–0.87]). Most of the stones concerned the submandibular gland (n = 24), mainly in the Wharton’s duct (19 stones), and 12 concerned the parotid glands; the concordance was considered to be good regarding the stone location (Kappa coefficient: 0.62 [95% CI 0.36–0.88]). The mean size of the stones was 7.54 ± 3.34 mm by 3D-CBCT sialography vs. 7.03 ± 3.48 mm by MR-sialography (p = 0.04).

Two patients presented with multiple stenosis. Most of the stenosis were in the parotid gland (n = 10) and only one involved the submandibular gland. Only one of these abnormalities was detected by both imaging procedures.

Dilatation was found in the submandibular gland in 14 cases and in the parotid gland in 11 cases. Most of the dilatations were classified as D1, with a mean diameter of 3.34 ± 0.97 mm as measured by 3D-CBCT sialography vs. 3.30 ± 1.25 mm by MR sialography (p = 0.57).

3D-CBCT sialography allowed exploration of the ductal system up to branch 4.25 ± 1.55 vs. 3.65 ± 0.96 by MR sialography of the submandibular gland (p = 0.14). In the parotid gland, the exploration reached branch 4.36 ± 1.41 with 3D-CBCT sialography vs. 4.08 ± 1.23 with MR sialography (p = 0.30). Ostium catheterization failed in two patients (4.3%) with 3D-CBCT sialography. The mean DAP generated by CBCT was 247.4 ± 76.7 mGy.cm^2^. We did not observe any adverse effects of either catheterization or iodine contrast injection.

## Discussion

Multiple imaging techniques are used for diagnosing salivary ductal diseases. Ultrasonography is a first-line examination as it exposes excellent diagnostic capabilities, with high sensitivity (0.899) and NPV (0.808) for exploration of the main salivary pathologies [30]. However, ultrasound lacks the capacity to identify the structures behind the bony structures, resulting in difficulties in exploration of the anterior part of the Wharton’s duct, it does not provide a representation of the salivary ducts, and it often fails to diagnose stones less than 2 mm in size [31–34]. CT scan, or its less irradiating CBCT counterpart, has a higher specificity than ultrasonography for determination of the number, the size, and the location of sialolithiasis [35, 36]. However, it fails to reveal radiolucent calculus, which can account for 10–20% of all sialolithiasis [11]. 3D-CBCT sialography is a relevant imaging modality as it allows accurate imaging and provides information regarding the catheterization of the salivary ostium and regarding the predicted diameter of the main duct. MR sialography has been validated as a suitable technique for evaluation of the ductal system of the salivary glands, especially for sialolithiasis and stenosis [11, 22, 37]. MR sialography is nevertheless not yet widely used and its relevance in the diagnostic strategy of salivary ductal pathologies remains to be fully elucidated.

Our study aimed to compare the diagnostic capability of 3D-CBCT sialography versus that of MR sialography. Our study population was comparable to those in previous publications in terms of age, gender distribution, and symptoms [18, 34]. Most patients had salivary symptoms for more than three months, this is due to the long tolerance of the obstructive symptoms including colic pain and swelling which are most often resolved at the end of the meals. Both imaging modalities allowed identification of the main salivary ductal diseases, with high positive percent agreement (> 0.8) for sialolithiasis, and good diagnostic performances for ductal dilatations. These results were supported by the high level of IOA (> 0.90) for visualization of ductal diseases. In a comparative study of MR sialography versus conventional sialography in 24 patients, Jäger et al. reported a similar sensitivity (0.93) and NPV (0.83) obtained with T2-weighted TSE for the detection of sialolithiasis [11]. Furthermore, the authors obtained 100% sensitivity and NPV with T2-weighted 3D constructive interference in steady-state (CISS) sequences; this MR modality was not performed in our study. Varghese et al. reported lower sensitivity (69%) for the detection of calculus disease in a study of 49 patients explored using MR sialography with T2-weighted TSE sequences [21]. In our study, 3D-CBCT sialography failed to diagnose sialolithiasis in four patients, with these conditions being located in the main duct of the parotid gland, while sialography allowed exploration up to the 4th -order branches in the same patients. In the absence of a complementary reference examination (sialoendoscopy), it was difficult to draw conclusions regarding false negatives; furthermore, salivary stones are less frequent in this location compared to the submandibular gland [38]. In addition, sialography is able to diagnose both radiopaque and radiolucent stones by appearing as a negative image in the contrast medium [28]. MR sialography was more effective at visualizing dilatations than 3D-CBCT sialography, as the technique is based on principles of MR hydrography, whereby T2-weighted pulse sequences are used to image static fluid (i.e., saliva) [19, 37]. Our results are consistent with those of other studies that have shown the ability of MR sialography to detect ductal dilatations [19–21]. 3D-CBCT sialography may be compromised in case of large sialoceles when 1 mL of contrast medium is not sufficient to fill all the salivary dilatations. With regard to stenosis, neither procedure was able to accurately identify these conditions, as evidenced by the low positive percent agreement (0.20). This result raises the question of the radiological definition of a salivary stricture, as evidenced by the lower IOA for identification of strictures (0.90) than sialolithiasis and dilatations. In a comparative study of conventional versus 3D-CBCT sialography, Jadu et al. reported the limited ability of 3D-CBCT sialography to identify salivary stenosis, especially in case of multiple lesions [16]. In their study, Varghese et al. reported very good outcomes with CISS sequences in MR sialography for the identification of strictures [21].

Regarding the last salivary branch of division visualized, 3D-CBCT sialography outperformed MR sialography irrespective of the gland studied. Many authors have reported that it is possible to explore up to the fifth-order branches with 3D-CBCT sialography [15–17, 28]. Nevertheless, the presence of a proximal obstruction (i.e., close to the salivary ostium) may prevent upstream opacification and, therefore, visualization of the proximal ducts. For MR sialography, the literature is concordant regarding the ability to explore up to the second branches of intra-glandular division, with visualization of the proximal branches not impaired by the presence of a ductal obstruction [11, 19]. MR sialography suffers from some limitations, including poor spatial resolution when using TSE or T2-weighted 3D gradient-echo sequences. Furthermore, the acquisition time is too long to preclude artifacts due to swallowing or head movements [11]. 3D-CBCT sialography results in exposure to a significant level of irradiation. Our study reported a mean DAP of 247.4 mGy.cm^2^, which is three times higher than the mean of 79.9 mGy.cm^2^ generated by panoramic radiography [39]. Nevertheless, by modulation of the exposure parameters, particularly regarding the reduced field of view, the mean DAP was lower than the mean of 1081−1162 mGy.cm^2^ reported for jaw imaging with different CBCT devices [39, 40]. Jadu et al. reported a similar effective radiation exposure with CBCT sialography and plain radiograph irrespective of the gland studied by using a 6-inch field of view and X-ray tube settings of 80 kVp and 10 mA [41]. Duct cannulation requires an experienced operator and can be compromised in patients with a low salivary flow or in case of stenosis of the papilla [19]. We observed only two cases of catheterization failure (4.3%) while the literature reports higher rates of failure of catheterization of 14–15% [18, 28]. The potential complications of sialography also include ductal trauma, displacement of calculus, infection, and reaction to the iodinated contrast medium [28, 42].

Our study suffers from some drawbacks. The first lies in our per-protocol analysis whereby we excluded patients in which we experienced a catheterization failure or discordant data regarding the side analyzed for the study. These side errors occurred in patients with multifocal symptoms. The MR images were acquired on a single gland in the sagittal plane, i.e., in the plane of the salivary ducts for better resolution, and they did not allow identification of the affected gland. The second is related to the presence of a high number of normal radiological examinations (n = 16/46), which could affect the diagnostic performances of the radiological procedures studied. There was no attempt to eliminate the “normal” observations from our analysis as they reflect the current practice. A third drawback, namely measurement bias, is possible in this type of imaging study, although it was limited by the double reading of the images by experienced radiologists and by the calibration session before starting the study analyses. Finally, it is regrettable that we did not compare our two imaging procedures with a reference imaging modality to decide discordant cases. Sialoendoscopy could represent such a positive control in further studies but may fail in cases of very proximal diseases. Another strategy is represented by the development of a consensus between radiologists and surgeons to establish a final diagnosis [11]. In the absence of a gold standard procedure, the term “sensitivity” and “specificity” has been replaced by the “positive percent agreement” representing the performance of an examination compared to the other one [16]. Finally, the absence of a definite diagnosis makes it difficult to determine the number of false-negative cases and, therefore, overestimates the positive percent agreement compared to the actual sensitivity of the examination.

## Conclusions

Notwithstanding the limitations of the study and the absence of statistical differences between the two imaging modalities, MR sialography may be more effective than 3D-CBCT sialography for the identification of sialolithiasis and ductal dilatations. Moreover, this non-invasive procedure is not subject to the failures and adverse effects of salivary catheterization. Both procedures allow exploration of the ductal system at least up to the third or fourth branch of division, irrespective of the study of the parotid or submandibular gland, and warrant being part of the diagnostic arsenal for non-tumorous salivary pathologies.

## Electronic supplementary material

Below is the link to the electronic supplementary material.

**Supplementary Material: Table 1**. STARD 2015 checklist for reporting diagnostic accuracy studies. **Table 2**. STROBE statement for reporting observational studies

## Data Availability

The datasets used and/or analyzed during the current study are available from the corresponding author on reasonable request.

## List of abbreviations

3D: Three-dimensional
CBCT: Cone-beam computed tomography
CI: Confidence interval
CISS: Constructive interference in steady-state
D: Dilatation
DRIVE: Driven equilibrium
FOV: Field of view
IG: Intra-glandular
IOA: Inter-observer agreement
L: Sialolithiasis
MIPMPR: Maximum Intensity ProjectionMultiplanar reconstruction
MRI: Magnetic resonance imaging
n: Number of patients
NPV: Negative predictive value
S: Stenosis
SD: Standard deviation
SE: Spin echo
pSS: Sjögren’s syndrome
SPIR: Spectral presaturation inversion recovery
TR/TE: Repetition/echo time
TSE: Turbo spin echo
