# Supplementary material for: A comparative study of three-dimensional cone-beam CT sialography and MR sialography for the detection of non-tumorous salivary pathologies
Source: BMC Oral Health. 2023 Jul 8;23:463. doi: 10.1186/s12903-023-03159-9 (PMC10329379; doi:10.1186/s12903-023-03159-9)
Supplement: Supplementary file 1 — Supplementary Material: Table 1. STARD 2015 checklist for reporting diagnostic accuracy studies. Table 2. STROBE statement for reporting observational studies [file 12903_2023_3159_MOESM1_ESM.docx]

**SUPPLEMENTARY MATERIAL**

#### Table 1. STARD 2015 checklist for reporting diagnostic accuracy studies

|  | **Section & Topic** | **No** | **Item** | **Reported on page #** |
| --- | --- | --- | --- | --- |
|  |  |  |  |  |
|  | **TITLE OR ABSTRACT** |  |  |  |
|  |  | **1** | Identification as a study of diagnostic accuracy using at least one measure of accuracy  (such as sensitivity, specificity, predictive values, or AUC) | #3 |
|  | **ABSTRACT** |  |  |  |
|  |  | **2** | Structured summary of study design, methods, results, and conclusions  (for specific guidance, see STARD for Abstracts) | #3 |
|  | **INTRODUCTION** |  |  |  |
|  |  | **3** | Scientific and clinical background, including the intended use and clinical role of the index test | #4 |
|  |  | **4** | Study objectives and hypotheses | #5 |
|  | **METHODS** |  |  |  |
|  | *Study design* | **5** | Whether data collection was planned before the index test and reference standard  were performed (prospective study) or after (retrospective study) | #6 |
|  | *Participants* | **6** | Eligibility criteria | #6 |
|  |  | **7** | On what basis potentially eligible participants were identified  (such as symptoms, results from previous tests, inclusion in registry) | #6 |
|  |  | **8** | Where and when potentially eligible participants were identified (setting, location and dates) | #10 |
|  |  | **9** | Whether participants formed a consecutive, random or convenience series | #6 |
|  | *Test methods* | **10a** | Index test, in sufficient detail to allow replication | #6-7 |
|  |  | **10b** | Reference standard, in sufficient detail to allow replication | #6-7 |
|  |  | **11** | Rationale for choosing the reference standard (if alternatives exist) | #8 |
|  |  | **12a** | Definition of and rationale for test positivity cut-offs or result categories  of the index test, distinguishing pre-specified from exploratory | #7-8 |
|  |  | **12b** | Definition of and rationale for test positivity cut-offs or result categories  of the reference standard, distinguishing pre-specified from exploratory | #7-8 |
|  |  | **13a** | Whether clinical information and reference standard results were available  to the performers/readers of the index test | #7 |
|  |  | **13b** | Whether clinical information and index test results were available  to the assessors of the reference standard | #7 |
|  | *Analysis* | **14** | Methods for estimating or comparing measures of diagnostic accuracy | #8 |
|  |  | **15** | How indeterminate index test or reference standard results were handled | #8 |
|  |  | **16** | How missing data on the index test and reference standard were handled | na |
|  |  | **17** | Any analyses of variability in diagnostic accuracy, distinguishing pre-specified from exploratory | na |
|  |  | **18** | Intended sample size and how it was determined | #8 |
|  | **RESULTS** |  |  |  |
|  | *Participants* | **19** | Flow of participants, using a diagram | #10 |
|  |  | **20** | Baseline demographic and clinical characteristics of participants | #11 |
|  |  | **21a** | Distribution of severity of disease in those with the target condition | #11 |
|  |  | **21b** | Distribution of alternative diagnoses in those without the target condition | na |
|  |  | **22** | Time interval and any clinical interventions between index test and reference standard | #11 |
|  | *Test results* | **23** | Cross tabulation of the index test results (or their distribution)  by the results of the reference standard | #14 |
|  |  | **24** | Estimates of diagnostic accuracy and their precision (such as 95% confidence intervals) | #12 |
|  |  | **25** | Any adverse events from performing the index test or the reference standard | #13 |
|  | **DISCUSSION** |  |  |  |
|  |  | **26** | Study limitations, including sources of potential bias, statistical uncertainty, and generalisability | #18 |
|  |  | **27** | Implications for practice, including the intended use and clinical role of the index test | #16-18 |
|  | **OTHER INFORMATION** |  |  |  |
|  |  | **28** | Registration number and name of registry | #6 |
|  |  | **29** | Where the full study protocol can be accessed | #6 |
|  |  | **30** | Sources of funding and other support; role of funders | #21 |
|  |  |  |  |  |

#### Table 2. STROBE statement for reporting observational studies

|  | Item No | Recommendation | Page No |
| --- | --- | --- | --- |
| **Title and abstract** | 1 | (*a*) Indicate the study’s design with a commonly used term in the title or the abstract | #3 |
|  |  | (*b*) Provide in the abstract an informative and balanced summary of what was done and what was found | #3 |
| Introduction | | | |
| Background/rationale | 2 | Explain the scientific background and rationale for the investigation being reported | #4 |
| Objectives | 3 | State specific objectives, including any prespecified hypotheses | #5 |
| Methods | | | |
| Study design | 4 | Present key elements of study design early in the paper | #6 |
| Setting | 5 | Describe the setting, locations, and relevant dates, including periods of recruitment, exposure, follow-up, and data collection | #6 and #10 |
| Participants | 6 | (*a*) Give the eligibility criteria, and the sources and methods of selection of participants. Describe methods of follow-up | #6 |
|  |  | (*b*) For matched studies, give matching criteria and number of exposed and unexposed | na |
| Variables | 7 | Clearly define all outcomes, exposures, predictors, potential confounders, and effect modifiers. Give diagnostic criteria, if applicable | #6-8 |
| Data sources/ measurement | 8* | For each variable of interest, give sources of data and details of methods of assessment (measurement). Describe comparability of assessment methods if there is more than one group | #6-8 |
| Bias | 9 | Describe any efforts to address potential sources of bias | #8 |
| Study size | 10 | Explain how the study size was arrived at | #8 |
| Quantitative variables | 11 | Explain how quantitative variables were handled in the analyses. If applicable, describe which groupings were chosen and why | #8 |
| Statistical methods | 12 | (*a*) Describe all statistical methods, including those used to control for confounding | #8 |
|  |  | (*b*) Describe any methods used to examine subgroups and interactions | na |
|  |  | (*c*) Explain how missing data were addressed | #10 |
|  |  | (*d*) If applicable, explain how loss to follow-up was addressed | #10 |
|  |  | (*e*) Describe any sensitivity analyses | #8 |
| Results | | |  |
| Participants | 13* | (a) Report numbers of individuals at each stage of study—eg numbers potentially eligible, examined for eligibility, confirmed eligible, included in the study, completing follow-up, and analysed | #10 |
|  |  | (b) Give reasons for non-participation at each stage | #10 |
|  |  | (c) Consider use of a flow diagram | #10 |
| Descriptive data | 14* | (a) Give characteristics of study participants (eg demographic, clinical, social) and information on exposures and potential confounders | #11 |
|  |  | (b) Indicate number of participants with missing data for each variable of interest | #10 |
|  |  | (c) Summarise follow-up time (eg, average and total amount) | na |
| Outcome data | 15* | Report numbers of outcome events or summary measures over time | #12-13 |

| Main results | 16 | (*a*) Give unadjusted estimates and, if applicable, confounder-adjusted estimates and their precision (eg, 95% confidence interval). Make clear which confounders were adjusted for and why they were included | #12 |
| --- | --- | --- | --- |
|  |  | (*b*) Report category boundaries when continuous variables were categorized |  |
|  |  | (*c*) If relevant, consider translating estimates of relative risk into absolute risk for a meaningful time period |  |
| Other analyses | 17 | Report other analyses done—eg analyses of subgroups and interactions, and sensitivity analyses | na |
| Discussion | | | |
| Key results | 18 | Summarise key results with reference to study objectives | #16 |
| Limitations | 19 | Discuss limitations of the study, taking into account sources of potential bias or imprecision. Discuss both direction and magnitude of any potential bias | #18 |
| Interpretation | 20 | Give a cautious overall interpretation of results considering objectives, limitations, multiplicity of analyses, results from similar studies, and other relevant evidence | #16-18 |
| Generalisability | 21 | Discuss the generalisability (external validity) of the study results | #18 |
| Other information | | | |
| Funding | 22 | Give the source of funding and the role of the funders for the present study and, if applicable, for the original study on which the present article is based | #21 |
